# Trump, Brexit, Right-wing Anti-globalisation, and An Uncertain Future for Public Health

**DOI:** 10.3934/publichealth.2017.2.139

**Published:** 2017-04-18

**Authors:** Isabelle Macgregor-Bowles, Devin C. Bowles

**Affiliations:** 1 Faculty of Health, University of Canberra, ACT, Australia; 2 Research School of Population Health, Australian National University, ACT, Australia

**Keywords:** Trump, Brexit, globalisation, anti-globalisation, international trade, public health, xenophobia, militarism, climate change

## Abstract

Global public health is intimately linked with political, economic and social determinants. The current global order has been built on the assumption that the globalisation agenda shared by political elites of the last several decades will continue. Individuals, businesses and countries have all made decisions, many of them linked to health, based on this assumption. The election of Donald Trump to the US presidency and the vote in Britain to exit the European Union exemplify a recent wave of right-wing anti-globalisation, which has risen in much of the West. The right-wing anti-globalisation movement will substantially affect global health through four pathways. Restrictions on trade will dampen economic growth and could diminish food security and the availability of medical supplies. Xenophobia will harm mental health through the lived experience of minorities, and will elevate the risk of economic and military conflict between countries. Increased defence expenditure in a time of limited government budgets will constrict funding available for healthcare and the social determinants of health. Mistrust of international treaties, including for climate change, will undermine the Paris Agreement and hasten greenhouse gas emissions. Without rapid mitigation, climate change could devastate population health globally through a range of mechanisms, including diminished food security and increased violent conflict. These would amplify many of the other health effects of right-wing anti-globalisation. By emphasising the shared humanity of all people, population health offers an antidote to the narrow focus of right-wing anti-globalisation.

## 1. Introduction

As the two most consequential elections in the last year, if not the last decade, the 2016 US presidential vote and poll on Britain's place in the European Union can usefully be analysed together. Some commentators argue that they are rightfully seen as part of a larger trend of increasing nationalism and xenophobia in the West which rejects the liberal, globalising consensus that has held sway since World War Two [1]. This might be termed the right-wing anti-globalisation movement. This trend could already be seen, for those who knew where to look, in the rise of Alternative in Germany, Front Nationale in France [1], and the Freedom Party in Austria [2], and in the efforts to rid Eastern Europe of Syrian refugees. The core of supporters for Donald Trump, Brexit and right-wing anti-globalisation more generally are whites without a university education who are poorly equipped for an interconnected global job market. They have witnessed their families' fortunes stagnating or declining [1],[3], and view globalisation as a threat rather than a promise.

These two elections, and the broader trend they represent, will likely be one of the new century's most important developments for global public health. At the time of writing, the full consequences of both elections are not yet known. Trump's presidency is still in its very early stages. Many of his pre-election rhetoric will be difficult or impossible to implement, not least because of the US Constitution. It is difficult to predict exactly what will come of his presidency. Similarly, the British Parliament has only just voted to begin the process of withdrawing from the European Union, and the ensuing negotiations will define the new relationship between Britain and the European Union. Some of the specific projections in this article may therefore not come to pass. They represent a larger truth, however, which is that the world has worked on the assumption that globalisation will continue to progress in the manner agreed almost unanimously by global elites for decades up to this point [4]. Food transport, trade agreements and military alliances are all based on this premise. Trump's election and Brexit portend a very different world, and undermine the expectations used in the calculations, made by the governments, businesses and individuals, that have delivered these arrangements. Global health will almost certainly suffer from this change.

Bypassing analysis of how a Trump presidency will likely decrease the availability of health insurance for millions of Americans, assuming he is able to get a bill through Congress, there are at least four major pathways through which the right-wing anti-globalising trend will affect public health. Increased protectionism and trade barriers which are likely with both Trump's presidency and Brexit could undermine food security and the availability of health and medical equipment and care. Increased xenophobia and racism will harm public health through domestic and international pathways, including reduced receptivity to refugees and increased militarization. Trump's pledge to increase military spending while decreasing the protections enjoyed by America's allies will see a major increase in global military spending. In a time of constrained economic growth, this will decrease funds for healthcare and the social determinants of health. Trump's presidency will likely be disastrous for greenhouse gas mitigation. Britain appears likely to remain a leader on climate change over the medium term, but its desire to avoid welcoming refugees from Syria portends a future in which it attempts to insulate itself from the effects of climate change at the expense of other countries.

Brexit and Trump's election also need to be understood as providing momentum to right-wing anti-globalisation throughout the world, and especially Europe. This paper uses these two votes as a lens through which to see a larger trend. Some commentators have noted how Brexit has bolstered populism elsewhere in Europe [4]. Trump's stunning election victory has also heartened populists globally. Knock-on effects potentially include altered election outcomes in much of Europe over the coming years, but time will tell. Any future implosion of a Trump presidency could have the opposite effect, but only after the event.

Before continuing, it is important to define the trend that Brexit and Trump's election represent as closely as possible. Others have noted that a number of populist movements are gaining strength, with left-wing populism holding views fairly close to those of left-leaning mainstream parties a few decades ago [5]. Right-wing populism is gaining a new ascendency in the West [5]. It is this populism that is represented and exemplified by Trump's election and, to a lesser extent, Brexit. Key attributes of the general trend include: a continued reliance on capitalism as an economic model with a deemphasis on government regulation of domestic businesses; a lack of regard for the environment, either explicitly stated or inherent in an approach to economic activity which holds that continued growth is key and that this is best achieved through lack of government regulation on business, and; xenophobia which might be considered to be as racist or Islamophobic, with associated emphasis on military preparedness and security. These specific characteristics mean that the version of anti-globalisation examined in this paper is far from the only possible version of anti-globalisation. The conclusions drawn about this form of anti-globalisation in this paper are similarly specific, and should not be interpreted as an entry into the wider debate on globalisation and health. Other visions of a world with less globalisation, such as that outlined by Naomi Klein in which ecological harmony and human rights are paramount [6], would have health very different from Trump's vision.

## 2. Protectionism and Economic Decline

The theme of US jobs being sent overseas, and unchecked immigration increasing competition for remaining American jobs, was prominent in Trump's campaign. The promise of reversing these trends was central to his appeal. While his message was often short on policy detail, he has suggested imposing a 45 per cent tariff on Chinese goods [7]. This could easily spark a trade war, diminishing trade between the world's two largest economies [7]. Some have predicted that full-scale trade wars stemming from Trump's policies would result in 4.8 million fewer jobs than without these wars [7]. Trump also said he would renegotiate trade agreements with other countries [7]. In Britain, many of the same fears of job loss to “others” helped fuel the Brexit vote. Whatever the specifics of Brexit, it is likely that trade between Britain and the European Union will decline. If nothing else, Britain's exit from the European Union will complicate and likely slow trade agreements with third countries or blocks. The subsequent loss of global trade and productivity from a Trump presidency and Brexit would interfere with global health. Most fundamentally, protectionism would increase the risk of countries not freely exporting food, which has already occurred recently in response to decreases in food production [8]. Restrictions on trade will increase food insecurity [8]. Impediments to the free flow of medical supplies would similarly be deleterious to health.

Trump's campaign also promised to decrease taxes substantially, in part because of a belief that keeping the money in private hands will stimulate the economy better than government spending. The theme of resources being wasted by European Union administration was also present in the British referendum, though the vote did not have any direct consequences of taxation rates. Other parties with right-wing anti-globalisation platforms have also called for decreased taxes. This is somewhat counterintuitive insofar as poorer people pay less in tax and often receive greater government benefits. As an economic reform, it is unlikely to address the domestic gap between rich and poor or bolster economic growth. If Trump's policies were enacted in the US, it would raise the debt and deficit precipitously, leading to an additional 7.2 trillion to the national debt over a decade [9]. Already, ratings agency Fitch has indicated that Trump's lack of predictability and confrontational style intensify risk to the global economy [10]. Medical care and the social determinants of health all require resources. The economic decline stemming from protectionist policies would have serious ramifications for health. Protectionism is also linked with a worldview which sees the relationship between countries solely through the lens of competition.

The diversity of government-supported programs that support health systems or the determinants of health is daunting. It is impossible to predict which ones will be cut or have their budgets squeezed. It is likely that humanitarian aid, both public and private, would also decline. Already, Trump has signalled that America's foreign aid expenditure will decline [11].

## 3. Xenophobia

It would be hard to deny that xenophobia played a role in Trump's election, given the promise to build a wall between the US and Mexico and prevent all Muslims from entering America. Similarly, fear of the “other”, was a factor in the Brexit vote. Other anti-globalisation political parties tend to be similar, including Alternative in Germany, which is strongly against the large number of Muslim refugees that have recently entered Germany [12]. Indeed, anti-globalisation is predicated on keeping the foreign “other” at bay, though precisely how the “other” is defined, such as by race, religion or nationality, may vary.

Trump's efforts to ban immigrants including refugees from seven Muslim-majority countries demonstrate the speed and scale that future xenophobic policies may take. The effects of xenophobia on domestic health require little imagination. Those who are discriminated against are at greater risk of distress, low self-esteem and mental illness. Xenophobia can also lead to reduced access to jobs, medical care and the social determinants of health for minority groups. By denying minorities an equal opportunity to fully contribute to health and society, they rob a country of some of its best healthcare providers and civic leaders.

Internationally, xenophobia can poison relations between countries and contribute to economic or military conflict. At a time when there are more forcibly displaced people than ever before, over 65 million [13], xenophobia hardens the heart of countries that have the financial and other resources to accept these people. The response of many European countries to Syrian refugees is illustrative, since there can be no denying that Syrians are at grave risk if they remain in their country. Similarly, Australia's policy of sending refugees who arrive by boat offshore to detention centres with deplorable conditions [14] illustrates how harmful xenophobia can be to the health of those who have a right to seek protection under international law.

## 4. Military Spending

Increases in nationalism and xenophobia are often associated with greater militarism. Trump was very clear during the election campaign that, if elected, he would increase military spending by up to $90 billion a year[15] and his recent budget proposal sees an increase of $54 billion a year [16]. Many nationalist parties have a similar focus on their countries' military. The American case is especially interesting because Trump also indicated that he would ask America's allies to pay for the defence that America provides. Even more radically, he suggested whether or not America would come to the aid of its allies depended on how well they had helped America. He specifically mentioned NATO allies, undermining the bedrock of the geopolitical order since the end of the Second World War.

The first consequence of Trump's rhetoric may be that America's allies will begin to spend more on their own militaries. This would be a reasonable, almost predictable response if they can no longer feel assured that America will come to their aid if they are attacked. America's allies may feel that their potential adversaries are not convinced that America would honour its commitments, meaning that American alliances lose some of their deterrent value. In a worst-case scenario, this could lead to aggression against an American ally that would not have otherwise occurred. Each of these scenarios would heighten global military spending.

Trump's pledge to boost American military spending while backing away from commitments to allies may also raise questions about America's intention in the minds of potential adversaries. America already has more conventional military power than any other country, and a substantial nuclear arsenal. With fewer allies to defend, why would America need to increase its military capacity? One possible answer is to bolster wars of aggression. If this interpretation gains traction, it is likely that America's potential adversaries, as well as its allies, would boost defence spending.

Government and societal resources are limited at the best of times. The financial constraints that right-wing anti-globalisation's restrictions could bring would further tighten constraints on spending. If military spending escalates due to right-wing anti-globalisation and nationalism, then there will be fewer resources that can be devoted to healthcare systems and to the social determinants of health. This would be reflected in domestic budgets and perhaps be amplified in foreign aid. Trump has already indicated that boosting military expenditure will be budget neutral because of cuts to other areas, including foreign aid [11]. His proposal on discretionary spending sees a decrease in funding of 18% to the Department of Health and Human Services, 14% to the Education Department, and 31% to the Environmental Protection Agency [16]. In a world with escalating military expenditure, total foreign aid could decrease and shift from humanitarian aid to military aid to help secure allied governments [17]. Trump's budget proposal cuts funding to the State Department by 29% [16].

## 5. Climate Change

Climate change has been called the greatest threat to global health of the 21^st^ century [18]. The Paris Agreement provides hope that humanity may be able to avoid substantial climate change that imperils human health. Throughout his candidacy, Trump signalled that he would withdraw from the Paris Agreement, and this stance is unchanged in his presidency. This would be a blow to climate if only because America is the second largest greenhouse gas emitter. Perhaps more importantly, America's withdrawal could lead other countries to do the same, or minimise their efforts. The dislike of international climate and other environmental treaties is characteristic of right-wing anti-globalisation thinking, which mistrusts treaties as ceding national sovereignty. Trump's executive orders to enable construction of the Dakota Access and Keystone XL pipelines were a blow to efforts to reduce climate change [19]. Trump's appointment of Rex Tillerson, previously the CEO of Exxon Mobile, as secretary of state is also ominous, but probably not as catastrophic to the climate as his appointment of Scott Pruitt to head the Environmental Protection Agency. Pruitt is recently on record questioning the science of climate change and has collaborated with the oil industry to block EPA regulation [20].

Some environmental and health campaigners might take solace in the fact that Trump has also pledged to block the Trans-Pacific Partnership, given that it would include provisions limiting the sovereignty of countries to protect the environment and their citizen's health. Yet some argue that while such provisions are problematic, a government seeking to avoid them could do so, even while such agreements are in force, in part because of abundant loopholes and the lack of an enforcement mechanism [6].

The Brexit campaign was characterised by similar concerns about national sovereignty. Britain appears likely to continue its leadership on climate change at least through the medium term, despite the Brexit vote. Brexit is concerning from a climate change perspective, however, because it was designed in part to insulate Britain from Syrian and other refugees coming to Europe in historically large numbers. Some analysts have argued that the Syrian civil war was precipitated or exacerbated by climate change [21],[22]. Regardless, future climate change will likely produce climate migrants seeking refuge in Europe. If Britain effectively insulates itself from this effect of climate change, it could diminish the urgency of how it addresses climate change in the future. The world would suffer with the absence of Britain's leadership on climate change.

Climate change will harm public health through three broad mechanisms [23]. Primary effects of climate change operate directly on the human organism, and can include increased mortality from heat waves. Secondary effects are ecologically mediated, such as altered distribution of some vector-borne diseases. Tertiary effects are socially mediated, and include increased risk of food insecurity, poorly planned migration, and conflict [23].

The tertiary effects of climate change will interact with and amplify many of the causal mechanisms by which right-wing anti-globalisation will harm health. It will reduce economic growth [24]–[26], providing another impediment to revenues to spend on public health. Without mitigation, climate change will reduce total food production and make it more volatile in many parts of the developing world, undermining food security and health [27],[28]. This will be especially damaging if international trade in food is restricted by anti-globalisation. Climate change is widely predicted to increase migration, especially within and between developing countries [29],[30]. This could lead to increased xenophobia [31],[32], especially in conjunction with right-wing anti-globalisation. In turn, this would impede rational decision making about migration, amplifying current trends in which developed countries deny entry to legitimate refugees [31], leading to further stress on alternative host destination health and social systems [32]. Finally, climate change has been predicted to harm health by increasing conflict [33]. Even without right-wing anti-globalisation, this would likely lead to increased militarisation with concomitant reductions in health and social spending [17]. In a context of rising right-wing anti-globalisation sentiments, the extent of militarisation is likely to increase.

## 6. Conclusion

Global public health relies on the smooth functioning of the political and economic order. This order, in turn, was based on the widespread assumption that the globalisation agenda will continue. This is reflected in everything from agricultural systems and food prices to military alliances. The rise of right-wing anti-globalisation, as reflected in Trump's election and Brexit, will require substantial adjustments to the political and economic order. These adjustments will have globally significant consequences for health.

The discipline of public health focuses on the health of all people, irrespective of their nationality, race, religion or gender. At its core, it ignores the dividing lines of borders, even if political analysis is required to further its aims. Public health's outlook is truly global, and it could therefore serve as a small, but important, counterweight to the restricted focus of the current right-wing anti-globalisation movement.
